# RATA: A method for high-throughput identification of RNA bound transcription factors

**DOI:** 10.14440/jbm.2017.171

**Published:** 2017-03-16

**Authors:** Karyn Schmidt, Frank Buquicchio, Johanna S. Carroll, Robert J. Distel, Carl D. Novina

**Affiliations:** 1 Department of Cancer Immunology and Virology, Dana-Farber Cancer Institute, Boston, MA 02215, USA; 2 Department of Medicine, Harvard Medical School, Boston, MA 02115, USA; 3 Broad Institute of Harvard and MIT, Cambridge, MA 02142, USA; 4 Belfer Office for Dana-Farber Innovations, Dana-Farber Cancer Institute, Boston, MA 02215, USA

**Keywords:** long non-coding RNA, transcription factor, scaffold, MS2 coat protein, RNA immunoprecipitation, ribonucleoprotein complex

## Abstract

Long non-coding RNAs (lncRNAs) regulate critical cellular processes and their dysregulation contributes to multiple diseases. Although only a few lncRNAs have defined mechanisms, many of these characterized lncRNAs interact with transcription factors to regulate gene expression, suggesting a common mechanism of action. Identifying RNA-bound transcription factors is especially challenging due to inefficient RNA immunoprecipitation and low abundance of many transcription factors. Here we describe a highly sensitive, user-friendly, and inexpensive technique called RATA (RNA-associated transcription factor array), which utilizes a MS2-aptamer pulldown strategy coupled with transcription factor activation arrays for identification of transcription factors associated with a nuclear RNA of interest. RATA requires only ~5 million cells and standard molecular biology reagents for multiplexed identification of up to 96 transcription factors in 2–3 d. Thus, RATA offers significant advantages over other technologies for analysis of RNA-transcription factor interactions.

## BACKGROUND

Dysregulation of lncRNAs has recently been linked to multiple human diseases, especially cancer [1]. While the exact mechanism of many lncRNAs remains unknown, it is becoming increasingly clear that many lncRNAs regulate gene expression through a variety of mechanisms, including direct association with transcription factors to regulate transcription factor activity and/or sub-cellular localization [2]. Therefore, technologies that interrogate the fundamental biology of lncRNAs, especially interactions with transcription factors, could accelerate biological discovery across many disciplines.

Unbiased identification of RNA-bound transcription factors following RNA immunoprecipitation (RNA-IP) is challenging for several reasons: (1) RNA-IPs are generally inefficient, requiring large numbers of cells to generate sufficient eluate for downstream analysis; (2) many transcription factors are expressed at very low levels and (3) the standard technique for unbiased identification of associated proteins is mass spectrometry, which requires a minimum threshold level of protein for detection. For example, the recent methods of ChIRP–MS [3] and RAP–MS [4] comprehensively identify proteins that interact with endogenous RNAs in cells through crosslinking of protein-RNA interactions and precipitation of endogenous RNAs by capture of biotinylated antisense DNA oligos. Due to low purification efficiency, these protocols require 100–800 million cells for each condition tested in order to generate sufficient eluate for mass spectrometry analysis. Thus, these protocols are costly and labor-intensive and may prohibit many scientists from performing them. Alternatively, other methods increase protein detection by increasing RNA purification efficiency. For example, incubation of *in vitro* transcribed, biotinylated RNA with cell lysate significantly increases efficiency of RNA purification [5]. However, because this method bypasses normal processing of endogenous RNAs, it may capture non-physiological protein-RNA interactions resulting from addition of RNAs post-lysis.

To overcome these obstacles, we developed a sensitive and unbiased technique for identifying RNA bound transcription factors called RATA [6]. This technique couples an RNA-IP with a high-throughput transcription factor activation array, enabling identification of up to 96 transcription factors in a single assay. Briefly, a mammalian cell line is co-transfected with plasmids encoding a nuclear localized FLAG-tagged MS2 protein and an RNA of interest tagged with the MS2 RNA aptamer. Following IP of the MS2-tagged RNA with anti-FLAG antibody, the immunoprecipitate is gently and efficiently eluted with FLAG peptide, retaining the native confirmation of any associated proteins. Finally, the eluted immunoprecipitate is directly input into a commercially available transcription factor activation array. Importantly, the IP is performed in parallel using cells transfected with MS2-tagged RNA or the untagged RNA control, enabling identification of transcription factors that are enriched in the RNA precipitate compared to the control precipitate. Because RATA amplifies the transcription factor signature through streptavidin-horseradish peroxidase, the amount of transcription factor required for detection is significantly reduced. Indeed, RATA sensitively and reproducibly enriches transcription factors from ~5 million cells (1/2 of a 10 cm dish). Furthermore, RATA requires only a plate reader for analysis, which is widely available to many scientists. Following cell harvest, only 2 to 3 days are required for analysis of up to 96 transcription factors.

While multiple methods exist for RNA-IP from cells, we have developed this technique using the well-established MS2-based system. The bacteriophage MS2 coat protein binds with high affinity (K_d_ of 7 nM) in a sequence-specific manner to a viral RNA stem-loop sequence (MS2 stem-loop) [7]. The V75E;A81G MS2 protein variant, selected for its lack of capsid formation in *E. coli*, has been widely adapted for localization and/or purification of tagged RNAs. In these approaches, a plasmid is generated to express the MS2 protein fused to a FLAG and/or GFP tag(s). The MS2 fusion expression plasmid, along with a second plasmid expressing an RNA engineered to contain multiple copies of the MS2 stem-loop structure, are transiently transfected into cultured cells. Highly-specific, *in vivo* binding of the MS2 fusion protein to the MS2-tagged RNA enables GFP-based localization or anti-FLAG IP of the RNA of interest [8,9]. The IP conditions used in this protocol are based on the MS2-IP protocol developed by Gong and Maquat, with several notable modifications [10]. First, we have designed an exclusively nuclear version of the MS2 fusion protein, enabling purification of nuclear RNA-associated transcription factors. Second, the IP is performed in the absence of any cross-linking. Third, to ensure protein compatibility with downstream transcription factor activation arrays, associated proteins and RNAs are gently eluted from beads using FLAG peptide.

Following FLAG peptide elution, the immunoprecipitate is directly input into a transcription factor activation array capable of monitoring multiple transcription factors simultaneously. This technology relies on a series of biotin-labeled DNA probes corresponding to consensus sequences of target transcription factors. When cellular lysate, or RATA immunoprecipitate, is incubated with this mixture of labeled probes, specific transcription factor/probe complexes will form. These protein/DNA complexes can be separated from unbound proteins and probes through spin-column separation. The retained probes are then released, hybridized to a plate in which each well is pre-coated with DNA complementary to a specific probe, and detected upon addition of horseradish peroxidase-conjugated streptavidin. While these arrays typically quantify activation (*i.e.* nuclear localization or expression) of transcription factors before and after a particular treatment, RATA uses the arrays to quantity the amount of transcription factors present in a control or RNA-specific IP.

High sensitivity, low cost, fast turnaround time, and the relative ease of use of RATA will allow scientists from a wide-range of disciplines to study RNA-transcription factor interactions. Although we have used RATA to identify transcription factors bound to lncRNAs, this protocol is compatible with other types of coding and non-coding RNAs. This is especially important as transcription factors have been shown to associate with a variety of RNAs implicated in many cellular processes and diseases, including non-coding and viral RNAs [11,12]. In summary, RATA offers significant advantages over existing technologies and is critical for scientists otherwise hindered from studying RNA-transcription factor interactions.

## MATERIALS

### Cells

A375 cells (ATCC, cat. # CRL-1619, Manassas, VA), or appropriate cell line.

### Reagents

#### Plasmid generation

- Ampicillin (Sigma-Aldrich, cat. # A5354, Dorset, UK)
- LB Broth, Miller (ThermoFisher Scientific, cat. # BP1426-500, Waltham, MA)
- pcDNA3.1 (-) + FLAG-NLS-MS2-eGFP (Addgene, plasmid ID 86827, Cambridge, MA)
- Plasmid maxi kit (QIAGEN, cat. # 12162, Hilden, Germany)

#### Cell culture and manipulation

- 100 mm Tissue Culture dishes (ThermoFisher Scientific, cat. # 08772E, Waltham, MA)
- 500 ml disposable vacuum storage unit, 0.2 μM pore, PES membrane (ThermoFisher Scientific, cat. # 09-761-107, Waltham, MA)
- Cell scraper, 1.8 cm blade, 25 cm handle (Medsupply Partners, cat. # TL-TR9000, Atlanta, GA)
- DMEM, high glucose, with pyruvate (ThermoFisher Scientific, cat. # 11995065, Waltham, MA)
- Fetal bovine serum (FBS) (ThermoFisher Scientific, cat. # 10437028, Waltham, MA)
- Lipofectamine 2000 Transfection Reagent (ThermoFisher Scientific, cat. # 11668027, Waltham, MA)
- PBS (phosphate buffered saline), 1 ×, pH ~7.4 (ThermoFisher Scientific, cat. # MT21040CV, Waltham, MA)

#### Immunoprecipitation

- cOmplete, Mini, EDTA-free Protease Inhibitor Cocktail (Sigma, cat. # 11836170001, St. Louis, MO)
- Dynabeads Protein G (ThermoFisher Scientific, cat. # 10003D, Waltham, MA)
- FLAG peptide (Sigma, cat. # F3290, St. Louis, MO)
- Low binding 1.7 ml microcentrifuge tubes, polypropylene (Westnet, cat. # 3207, Canton, MA)
- Monoclonal ANTI-FLAG M2 antibody (Sigma, cat. # F1804, St. Louis, MO)
- Normal rabbit IgG (Santa Cruz Biotechnology, cat. # sc-2027, Dallas, TX)
- Phenylmethanesulfonyl fluoride (PMSF) solution (Sigma, cat. # 93482, St. Louis, MO)
- RNaseOUT Recombinant Ribonuclease Inhibitor (ThermoFisher Scientific, cat. # 10777019, Waltham, MA)
- TF Activation Profiling Plate Array(s) (Signosis, Inc., cat. # FA-1001 – FA-1011, Santa Clara, CA)
- Tris HCl, molecular biology grade (ThermoFisher Scientific, cat. # BP153-500, Waltham, MA)
- Triton X-100, molecular biology grade (ThermoFisher Scientific, cat. # AC327371000, Waltham, MA)

#### Protein and RNA analysis

- 10 × Tris/Glycine/SDS Running Buffer (Bio-Rad, cat. # 1610732, Hercules, CA)
- 2 × Laemmli Sample Buffer (Bio-Rad, cat. # 1610737, Hercules, CA)
- Any kD Mini-PROTEAN TGX Precast Protein Gels, 10-well (Bio-Rad, cat. # 4569033, Hercules, CA)
- iScript cDNA synthesis kit (Bio-Rad, cat. # 1708891, Hercules, CA)
- Precision Plus Protein Kaleidoscope Prestained Protein Standards (Bio-Rad, cat. # 1610375, Hercules, CA)
- RNeasy Micro Kit (QIAGEN, cat. # 74004, Hilden, Germany)
- SsoAdvanced Universal SYBR Green Supermix (Bio-Rad, cat. # 1725270, Hercules, CA)

### Recipes

- 5 mg/ml FLAG stock solution in 1 × PBS
- Lysis buffer: 20 mM Tris HCl pH 7.4, 10 mM NaCl, 2 mM EDTA. Immediately prior to use, add detergent, and protease and RNase inhibitors as follows: 0.5% Triton X-100, 40 U/ml RNaseOUT, 1 mM PMSF, Roche protease inhibitor cocktail (– EDTA).
- Wash Buffer: 50 mM Tris pH 7.4, 200 mM NaCl. Immediately prior to use, add 0.05% Triton X-100.

### Equipment

–20°C freezer
–80°C freezer (optional)
- Bio-Rad CFX Connect machine and Bio-Rad CFX Manager 3.1 software (or any qPCR instrument and software)
- CO_2_ cell incubator
- DeNovix DS-11 (DeNovix, Wilmington, DE) or similar spectrophotometer
- Magnetic stand for 1.5 ml tubes
- Microplate reader with a 96-well microplate tray and associated software
- Refrigerated centrifuge
- Shaking 37°C incubator for bacterial culture
- Vertical Electrophoresis Cell for Protein Gels (such as Mini-PROTEAN Tetra Cell, Bio-Rad, cat. # 1658005, Hercules, CA)

## PROCEDURE

**Figure 1.**
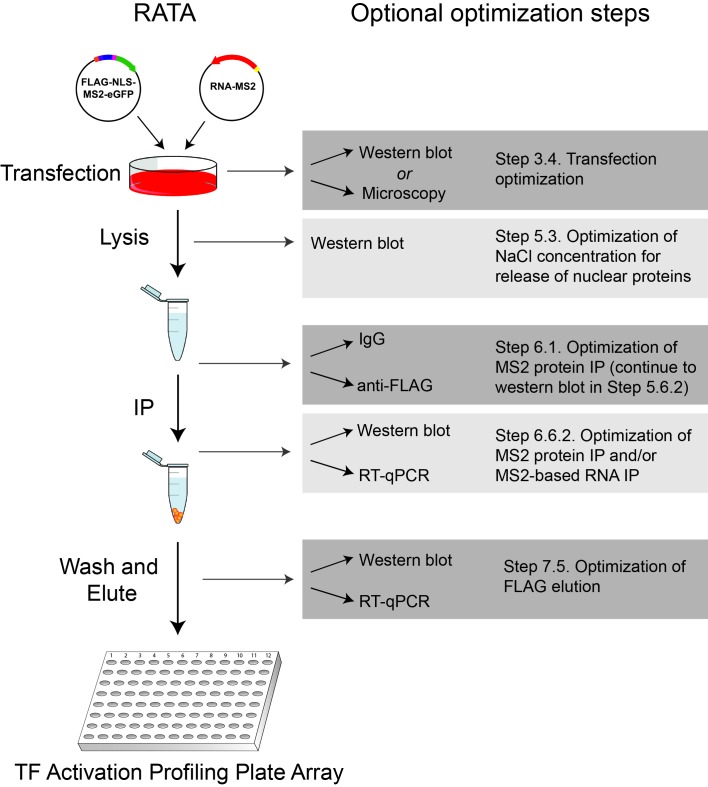
Flow diagram of RATA protocol. Optional optimization steps are denoted on the right with the corresponding step in the RATA protocol highlighted.

This protocol was optimized for the adherent A375 melanoma cell line. Expression, lysis and elution conditions may need to be optimized for each cell line, and steps requiring optimization are highlighted throughout this protocol and shown schematically in **Figure 1.** MS2-based IP of the RNA should be verified before completing the RATA protocol for an RNA of interest. To verify IP, complete the protocol through the washes at step 6.6.2, and split the eluate for western blot analysis of MS2 IP, and RT-qPCR analysis of RNA-IP. For all RNA isolations, lysate or eluate can be added directly to TRIzol^®^/TRIzol^®^ LS or QIAGEN’s buffer RLT, and RNA isolation should be completed according to manufacturer’s instructions.

### Plasmid generation

1. Design MS2-tagged and control expression vectors for RNA of interest
  **1.1.** Clone your RNA of interest into an appropriate mammalian expression vector (such as pcDNA3.1, Invitrogen) and confirm expression in your cell line of choice *via* RT-qPCR. This will serve as the untagged control vector.
  **1.2.** Insert 12 copies of the MS2 stem-loop RNA sequence at the 5’ or 3’ end of your RNA in the control vector. The MS2 stem-loop sequences may be cloned and inserted from preexisting vectors, such as Addgene plasmid # 27119 [8]. If possible, confirm that the insertion of the MS2 stem-loop sequences do not interfere with normal functionality of the RNA. For example, differential gene expression or phenotypic changes may be quantified following transfection of either the untagged or tagged RNA.
    **CAUTION:** Many lncRNA annotations include an AT-rich transcription termination sequence. Inclusion of this sequence upstream of the MS2 stem-loops may prematurely terminate transcription and exclude the MS2 stem-loop tag from the transcript. The RNA sequence of interest should be examined and, if present, the transcription termination sequence should be excluded from the MS2 expression vector. To ensure similar expression levels between the tagged and untagged transcripts, we recommend cloning the same sequence for expression of both the control and MS2-tagged RNA.
    **NOTE:** As with any screening methodology, the possibility of false positive hits should be considered. In RATA, false positive signals may result from non-specific interactions of transcription factors with the MS2 stem-loops. To account for this possibility, we recommend performing a control RATA experiment using cells transfected with the MS2 fusion expression plasmid and either an empty or MS2 stem-loop expression plasmid, both lacking the RNA of interest. This will identify any transcription factor signals resulting from non-specific interactions with the MS2 stem-loop RNA tag. False positive signals may be reduced by optimizing the RATA protocol (for example, decreasing the amount of plasmid used for cell transfection). Additionally, signals from the control RATA experiment may be subtracted from RATA results using MS2 stem-loop expressing plasmid containing the RNA of interest.
2. Prepare plasmids
  **2.1.** Culture *E. coli* containing RNA or MS2 expression plasmids overnight in a shaking incubator at 37°C in LB with ampicillin (100 µg/ml) or correct antibiotic for selection.
  **2.2.** Purify plasmids using QIAGEN’s plasmid maxi kit according to manufacturer’s instructions.
  **2.3.** Measure DNA concentration and purity using DeNovix.

### Preparation of mammalian cell extract

All reagents and materials used during tissue culture manipulation should be used in a sterile tissue culture hood equipped with ultra-violet light for decontamination. Nuclear localization of the MS2 protein should be confirmed for each new cell line *via* fractionation and western blot analysis, or *via* microscopy.

**3.** Transfect mammalian cells
  **3.1.** Seed cells at ~40% confluency (cell number depends on cell type) in 10 cm dishes in appropriate media (such as DMEM + 10% FBS).
  **3.2.** Incubate cells at 37°C in a humidified incubator, 5% (v/v) CO_2_, for at least 8 h, until cells reach ~70%–80% confluency.
  **3.3.** Change cell medium.
  **3.4.** Transiently co-transfect cells using Lipofectamine 2000 according to manufacturer’s protocol, with the FLAG-NLS-MS2-eGFP plasmid, and the lncRNA expression plasmid (either MS2-tagged or an untagged control).
    **TIP:** (optimization): Transfection conditions may need to be optimized for each cell line. Cells should be transfected with varying amounts of each plasmid, grown for 24–72 h, and expression of the RNA and FLAG-NLS-MS2-eGFP fusion protein should be quantified by RT-qPCR and western blot analysis, respectively. Optimal expression in the melanoma cell line A375 was observed using 10 µg of the FLAG-NLS-MS2-eGFP plasmid and 8 µg of the lncRNA expression plasmids, 48 h. post-transfection.
  **3.5.** Grow cells for 24–74 h.
**4.** Harvest cells
  **4.1.** Remove media, and rinse cells in 3 ml 1 × PBS.
  **4.2.** Detach cells by scraping into 5 ml of ice cold 1 × PBS.
    **NOTE:** Cells may also be detached by trypsinization (incubate cells with ~3 ml of trypsin for ~5 min or until cells detach, transfer to 15 ml conical tube with ~7 ml of media, and collect cells by centrifugation at 300 × g for 3 min) followed by an additional rinse in 5 ml of 1 × PBS.
  **4.3.** Collect cells by spinning at 300 × g for 3 min, remove PBS.
    **TIP:** Cells may also be snap-frozen (using liquid nitrogen or crushed dry ice) and stored at −80°C if not proceeding directly to cell lysis and IP.

### Cell lysis and IP

To prevent loss of RNA, we recommend the use of low-adhesion/retention tubes and pipette tips for all steps of IP.

**5.** Lyse cells
  **5.1.** Add 500 µl of cold lysis buffer. Pipette up and down to resuspend cells, and transfer to a 1.5 ml tube.
  **5.2.** Rotate at 4°C for 10 min.
  **5.3.** Add NaCl to a final concentration of 150 mM (15 µl of 5 M NaCl into 500 µl).
    **TIP:** (optimization): These lysis conditions do not completely disrupt the nucleus, but rather allow for ‘leaking’ of nuclear proteins. NaCl concentrations may need to be optimized for each cell line. To optimize NaCl concentration, divide equal volumes of cell suspensions into 1.5 ml tubes, add varying amounts of NaCl to each tube that covers a range between 150 mM to 300 mM final NaCl concentrations, and complete protein lysis. Western blot analysis and probing with an anti-FLAG antibody will confirm the concentration of NaCl required for maximal isolation of the FLAG-NLS-MS2-eGFP protein.
  **5.4.** Incubate on ice for 10 min, with occasional and gentle swirling.
  **5.5.** Centrifuge at 17000 × g at 4°C for 10 min, and transfer supernatant to new tube.
  **5.6.** Measure protein concentration on DeNovix.
  **5.7.** Save 2 × 25 µl input samples: one for RNA and one for protein analysis. For protein analysis, add 25 µl of 2 × Laemmli buffer and boil for 5 min at 95°C. For RNA analysis, add 350 µl QIAGEN buffer RLT and complete RNA isolation according to manufacturer’s protocol.
    **NOTE:** RNA may also be isolated using TRIzol^®^ LS Reagent (Invitrogen, cat. # 10296010, Carlsburg, CA) following manufacturer’s instructions. The use of an RNA carrier (such as glycogen) may be required for enhancement of RNA recovery from low yield inputs.
**6.** Perform immunoprecipitation
  **6.1.** Add 2.5 µg ANTI-FLAG^®^ M2 antibody (Sigma) to cell lysate.
    **TIP:** (optimization): If optimizing MS2 protein IP, split cell lysate, and add ANTI-FLAG^®^ M2 antibody (Sigma) to one half, and matched IgG control antibody to the other half. This will allow optimization of MS2-based IP of the RNA of interest compared to the IgG non-specific control. The amount of antibody may need to be adjusted depending on the expression level of the fusion protein in your cell line of interest. Once RNA-IP is optimized, RATA should be completed from anti-FLAG IPs of lysates expressing either the tagged or untagged RNA.
  **6.2.** Rotate for 2 h at 4°C.
    **TIP:** IP may also be performed overnight.
  **6.3.** Prepare beads
    **6.3.1.** Pipette 2 × 25 µl protein-G beads (50% slurry) into two 1.5 ml low-adhesion tubes, add 500 µl of PBS, place tubes on magnet and remove supernatant.
    **6.3.2.** Wash 1 time with 500 µl 1 × PBS.
    **6.3.3.** Wash 1 time in 100 µl salt-supplemented cell lysis buffer.
  **6.4.** Add cell lysate to prepared beads.
  **6.5.** Rotate for 1 h at 4°C.
  **6.6.** Wash beads
    **6.6.1.** Remove supernatant.
    **TIP:** (optimization): If optimizing protein IP conditions (see step 6.1), save flow through samples (~25 µl) for protein analysis.
    **6.6.2.** Wash 5 times in 500 µl with MS2 low salt wash buffer.
    **TIP:** (optimization): If optimizing IP conditions, add 25 µl 2 × Laemmli buffer directly to beads following washed and boil for 5 min at 95°C. Remove samples from beads and split for protein and RNA analysis. For RNA analysis, add 350 µl of QIAGEN buffer RLT and complete RNA isolation following manufacturer’s instruction. Once IP has been confirmed, proceed directly to elution.
**7.** Elute RNA and associated proteins
  **7.1.** Prepare 100 µg/ml FLAG peptide in wash buffer by diluting 5 µl of FLAG stock solution (5 mg/ml in 1x PBS) into 250 µl wash buffer.
  **7.2.** Add 25 µl of FLAG peptide solution to lysate.
  **7.3.** Rotate for 30 min at 4°C.
  **7.4.** Remove and save eluate.
  **7.5.** Repeat steps 7.2 through 7.4 for a total of 2–3 elutions.
    **TIP:** (optimization): If performing the elution for the first time, RT-qPCR and western blot analysis should be performed to determine when optimal elution occurs. To determine elution efficiency, add 25 µl of 2 × Laemmli buffer directly to beads and boil for 5 min at 95°C. Remove samples from beads and split for protein and RNA analysis. The fraction containing the highest amount of RNA should be used for subsequent analysis in the TF Activation Profiling Plate Array. Alternatively, 2–3 elutions may be combined.
    **TIP:** Elution may be snap-frozen (using liquid nitrogen or crushed dry ice) and stored at –80°C if not proceeding directly to TF activation profiling plate array.
**8.** Perform TF Activation Profiling Plate Array
  **8.1.** Mix the following components in a low-adhesion 1.5 ml tube: 15 µl TF binding buffer (from kit), 3 µl TF Probe mix (from kit) and 12 µl Eluate (from step 7.5).
  **8.2.** Follow manufacturer’s instructions to complete the transcription factor array.
    **CRITICAL STEP:** The transcription factor activation array must be completed for both the tagged RNA-IP and the untagged RNA-IP negative control simultaneously in order to allow determine IP-specific transcription factor enrichments. The use of the untagged RNA control will account for any changes in expression (and therefore background signal) of any transcription factors affected by expression of the RNA.
  **8.3.** Following completion of the protocol and quantification of the signal from each well on a microplate reader, analyze the data as follows (and as exemplified in **Table 1**):
    **8.3.1.** Normalize raw values by calculating the ratio of each transcription factor specific signal over the signal of a non-specific probe (*i.e.* the signal corresponding to a transcription factor that is not bound by the RNA of interest). Transcription factor II D (TFIID) can be used in most cases.
    **8.3.2.** Calculate individual transcription factor enrichment by taking the ratio of the normalized signal of the specific (tagged) RNA-IP compared to the normalized signal of the control (untagged) RNA-IP.
    **NOTE:** To account for fluctuations in transcription factor levels between samples, we recommend repeating the assay for a total of 2–3 biological replicates to confirm specific signals.

## ANTICIPATED RESULTS

**Figure 2.**
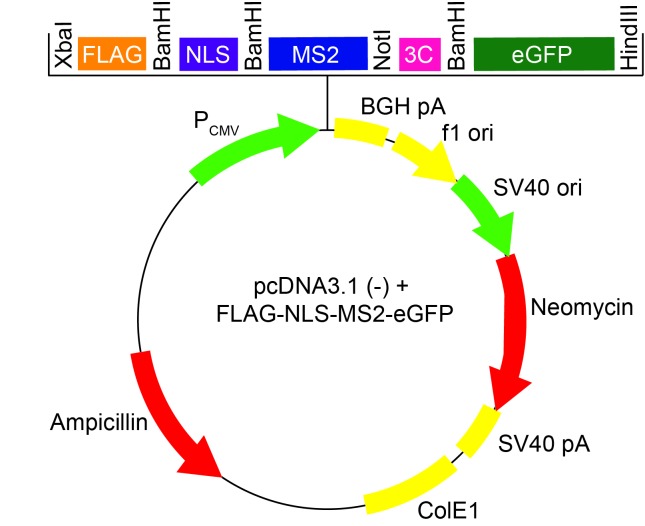
Schematic representation of the pcDNA3.1 (-) + FLAG-NLS-MS2-eGFP vector. The MS2 fusion protein is expressed from the pcDNA3.1 (-) constitutive CMV promoter. NLS = SV40 NLS sequence, 3C = human rhinovirus 3C protease cleavage site. Selected restriction enzymes sites within the inserted sequence are highlighted.

**Figure 3.**
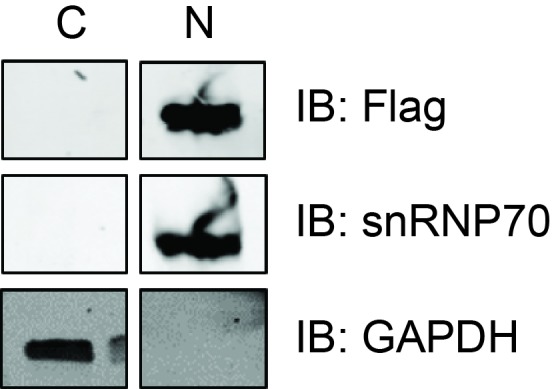
Western blot analysis confirms nuclear localization of MS2 fusion protein. A375 cells transfected with the pcDNA3.1 (-) + FLAG-NLS-MS2-eGFP vector were fractionated with the ThermoFisher Scientific NE-PER Nuclear and Cytoplasmic Extraction Kit, according to the manufacturer’s instructions. Cytoplasmic (C) and nuclear (N) fractions were subjected to western blot analysis. The blot was immunoblotted (IB) with monoclonal ANTI-FLAG M2 antibody (Sigma), α-Hsp90 4F10 antibody (Santa Cruz), and α-GAPDH 14C10 antibody (Cell Signaling Technology).

The protocol proved here offers low-cost, highly-sensitive identification of transcription factors associated with an RNA of interest. Our method uses a slightly modified MS2-based IP, which has successfully been used to purify RNAs, including lncRNAs [9,10]. We have designed a plasmid expressing an exclusively nuclear version of FLAG-tagged MS2-eGFP fusion protein (**Fig. 2**). While fractionation and western blot analysis of A375 cells transfected with this plasmid confirms nuclear localization of the FLAG-NLS-MS2-eGFP fusion protein (**Fig. 3**), nuclear localization of the fusion protein should be confirmed for each new cell line prior to RATA. As an alternative to cellular fractionation, localization may be verified through microscopy-based visualization of the eGFP tag.

**Figure 4.**
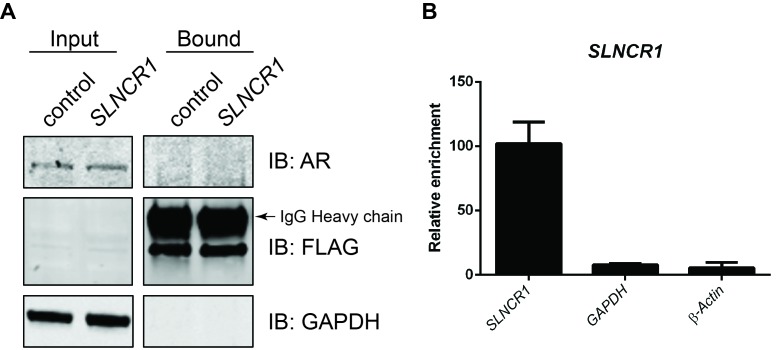
Western blot analysis fails to identify AR, a known *SLNCR1* interactor, after MS2-based RNA-IP. (**A**) A375 cells were transfected with 10 µg of the FLAG-NLS-MS2-eGFP expressing plasmid and 8 µg of an untagged (control) or 12 × MS2 binding site 3’ tagged *SLNCR1* expression vector. FLAG-tagged MS2 was immunoprecipitated from A375 using an anti-FLAG antibody, and total protein input or bound proteins was subjected to western blot analysis. The blot was probed with 1:200 α-androgen receptor (AR) N-20 (Santa Cruz Biotechnology), 1:5000 ANTI-FLAG M2 (Sigma) and 1:5000 α-GAPDH (Cell Signaling). The FLAG-NLS-MS2-eGFP protein has a predicted molecular weight of approximately 45 kDa, and is detectable below the IgG heavy chain located at approximately 50 kDa. (**B**) Relative enrichment of the indicated transcripts as measured by RT-qPCR compared to RNA enriched from cells expressing *SLNCR1* without MS2 stem-loops. Total proteins and RNAs were released by incubating in Laemmli buffer at 95°C for 5 min.

**Figure 5.**
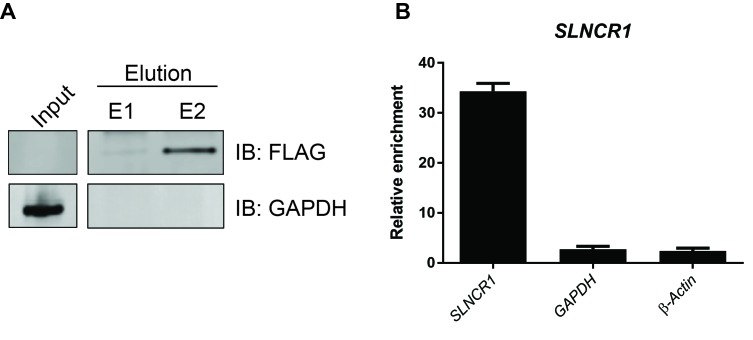
MS2-based IP and FLAG peptide elution significantly enriches the lncRNA *SLNCR1*. (**A**) A375 cells were transfected with 10 µg of the FLAG-NLS-MS2-eGFP expressing plasmid and 8 µg of a 12 × MS2 binding site 3’ tagged *SLNCR1* expression vector. FLAG-tagged MS2 was immunoprecipitated from A375 using an α-FLAG antibody, and bound proteins and RNAs were eluted by incubation with FLAG peptide for 30 min at 4°C. E1 refers to the first elution, E2 refers to a second elution from the same IP. Total protein input or eluted proteins were subjected to western blot analysis, and the blot was probed with 1:5000 ANTI-FLAG M2 (Sigma) and 1:5000 α-GAPDH (Cell Signaling). (**B**) Relative enrichment of the indicated transcripts from E2 as measured by RT-qPCR compared to RNA enriched from cells expressing *SLNCR1* without MS2 stem-loops.

Recently, we used RATA to identify transcription factors associated with the lncRNA *SLNCR1* (LINC00673) [6]. As shown in **Figure 3**, lysis conditions used in this protocol efficiently recover the nuclear fusion protein, and the associated MS2-tagged *SLNCR1* RNA is routinely enriched ~100-fold over an untagged control (**Fig. 4A** and **4B**). As opposed to recovery of bound proteins and RNAs through boiling in 2 × Laemmli buffer (**Fig. 4A** and **4B**), elution using FLAG peptide recovers slightly lower amounts of protein and RNA, usually between 5- and 40-fold enrichment of the tagged RNA (**Fig. 5A** and **5B**). Despite this lower recovery, RATA is sensitive enough to detect transcription factors present in eluate from FLAG peptide elution (**Table 1**) even when the transcription factor (in this example, the androgen receptor (AR)) is not detectable *via* western blot analysis of the MS2-tagged *SLNCR1* immunoprecipitate (**Fig. 4A**). In addition to being very sensitive, enrichments detected *via* RATA are highly-reproducible between biological replicates. For example, RATA of *SLNCR1* shows reproducible enrichment of Brn3a (25-fold in replicate 1, and 12-fold in replicate 2), AR (10-fold in replicate 1, and 8-fold in replicate 2), and PAX5 (8-fold in replicate 1, and 11-fold in replicate 2) (**Table 1** and data not shown). Thus, because levels of transcription factors may naturally fluctuate between samples, IP-specific signals may be confirmed through 2-3 biological replicates of RATA.

Because our methodology requires overexpression of the tagged RNA of interest, there are important potential caveats to consider when using this approach. First, inclusion of an RNA tag may affect secondary structure of the RNA, and therefore may also affect interactions with partner proteins. If possible, phenotypic or gene expression changes should be quantified following overexpression of either the untagged or tagged RNA to determine if the tag interferes with normal functionality of the RNA (Step 1.2.1). Ideal placement of the RNA tag may need to be determined empirically. Second, overexpression of RNA may increase/induce non-specific binding with proteins that do not interact with the RNA under physiological conditions. Thus, candidate RNA-transcription factor interactions identified through RATA should be validated through orthogonal assays. For example, RIP assays, in which an RNA binding protein of interest is immunoprecipitated and associated RNAs are identified through RT-qPCR, may be used to confirm if a candidate transcription factor interactor identified through RATA maintains its interaction with endogenous RNAs [13]. Indeed, RIP assays confirmed the two top *SLNCR1* interactors (Brn3a and AR) [6].

While RATA is limited to the identification of transcription factors included in the arrays, there are multiple versions of commercially available transcription factor activation arrays compatible with this technique. Signosis offers 12 different arrays, each including various combinations of over 150 unique transcription factors. Panomics offers 5 different transcription factor activation arrays (TranSignal™ Protein/DNA Array Systems), with their largest array (Combo Array) able to quantify 345 transcription factors in a single assay. Collectively, the Panomics and Signosis Arrays cover over 400 unique transcription factor in varying combinations. Importantly, these transcription factors represent the most biologically relevant and well-characterized transcription factors, covering approximately 25% of the 1672 currently identified human transcription factors [14]. Additionally, because the TranSignal™ Arrays rely on membrane-based chemiluminescent detection, with DNA probes spotted on a membrane rather than a 96-well plate, these arrays may be ideal for scientists who do not have access to a plate reader. For scientists with access to a Luminex machine, Panomics also offers two Luminex^®^ based assays capable of quantifying 40 transcription factors in up to 96 samples (such as controls, technical- and/or biological- replicates) in a single plate. The wide variety of transcription factor arrays available enables great flexibility in assay design. The selection of a transcription factor array depends on the RNA being studied and the type of information desired. For example, if a scientist is interested in testing the hypothesis that a lncRNA binds to transcription factors, Signosis’ Array I or II, which contain 48 or 96 of the most well-studied transcription factor, respectively (many of which have been shown to bind to lncRNAs), may be suitable. Alternatively, if the lncRNA in question is expressed exclusively in cancer and implicated in tumorigenesis, Signosis’ Cancer Stem Cell Array (containing 4 sets of 23 transcription factor specific probes) may be appropriate. For a more comprehensive analysis of associated transcription factors, scientists may choose to screen multiple arrays, or use the 345 probe Panomics Combo Array. It may also be helpful to consider which transcription factors are expressed in the cell line being tested; however, the arrays are very sensitive and are capable of detecting transcription factors even when they are not detected *via* western blotting.

This report details a novel protocol for the efficient and low cost identification of transcription factors associated with an RNA of interest, including lncRNAs. We have designed an exclusively nuclear MS2 fusion protein and optimized conditions to enable highly efficient isolation of ribonucleoprotein complexes. This easily implemented protocol will enable scientists from a range of fields to more efficiently and comprehensively investigate the role that RNAs play in modulating transcription factor activity and regulating gene expression.

## TROUBLESHOOTING

Potential problems and troubleshooting suggestions are listed in **Table 2**.

## Figures and Tables

**Table 1.** Example data obtained from RATA using the lncRNA *SLNCR1*.

	Transcription factor	Values	Normalized values	Fold enrichment
Control *SLNCR1* (no MS2-loops)	Brn-3	17421	0.18	
	AR	26840	0.27	
	Pax-5	178321	1.82	
	GR/PR	53194	0.54	
	NFAT	110426	1.12	
	SMAD	276121	2.81	
	MEF2	94463	0.96	
	GATA	98180	1.00	
MS2-tagged *SLNCR1*	Brn-3	122792	4.48	25.27
	AR	77925	2.85	10.41
	Pax-5	407732	14.89	8.20
	GR/PR	15177	0.55	1.02
	NFAT	25774	0.94	0.84
	SMAD	90559	3.31	1.18
	MEF2	19599	0.72	0.74
	GATA	27381	1.00	1

RATA was performed with A375 cell lysates transfected with pcDNA3.1 (-) + FLAG-NLS-MS2-eGFP and a vector expressing either the untagged or MS2-tagged *SLNCR1*. Eluate was subjected to the Signosis, Inc.’s TF Activation Profiling Plate Array I, and transcription factor names correspond to names in the array. Shown are signals for selected transcription factors selected from a signal, representative assay. Raw fluorescence values are shown in column 3, and normalized values are shown in column 4 (calculated as the ratio of the transcription factor specific signal over the GATA signal). Fold enrichment is calculated as the ratio of the normalized signal of MS2-tagged IP versus the normalized signal of the untagged control IP.

**Table 2.** Troubleshooting table.

Step	Problem	Cause	Suggestion
1.1	RNA is not expressed	Incompatible promoter in plasmid	Find an expression plasmid compatible with your cells
3.4	No expression of fusion MS2 protein, or RNA	Inefficient transfection	Check purity of plasmid DNA, and ethanol precipitate DNA if necessary Optimize DNA transfection conditions for cells
6.6	Failure to IP MS2 protein	MS2 protein is not being released from nucleus during lysis Insufficient antibody and/or dynabeads	Test multiple NaCl concentrations to find optimal concentration for release of nuclear MS2 protein Increase the amount of FLAG antibody and/or dynabeads
6.6	No enrichment of RNA in IP	MS2 stem-loop sequences are not included in mature transcript MS2 stem-loop sequences are blocked from binding to MS2 protein	Examine RNA sequence and remove AT-rich transcription termination sequence upstream of MS2 sequences (if 3’ tagged) Move RNA tag to the other end of the transcript
6.6	No detectable RNA	Presence of RNase(s) in one or more buffer causing RNA degradation	Use only nuclease-free reagents and buffers throughout procedure Filter buffers through a 0.2 μM filter to remove RNase(s)
6.6	RNA is only detectable from input samples	RNA concentration in IPs is too low for efficient isolation	If using a Trizol based RNA extraction, include an RNA carrier (such as glycogen) to increase RNA recovery
7.5	Failure to elute MS2 protein	Degradation of FLAG peptide	Remake FLAG peptide dilution in protease-free PBS Aliquot FLAG stock solution to avoid multiple freeze-thaws
8	Failure to enrich transcription factors	Overwashing is stripping transcription factors from ribonucleoprotein complexes	Reduce the number of washes, or reduce the level of detergent in wash buffer
